# Advances in Stem Cell Modeling of Dystrophin-Associated Disease: Implications for the Wider World of Dilated Cardiomyopathy

**DOI:** 10.3389/fphys.2020.00368

**Published:** 2020-05-12

**Authors:** Josè Manuel Pioner, Alessandra Fornaro, Raffaele Coppini, Nicole Ceschia, Leonardo Sacconi, Maria Alice Donati, Silvia Favilli, Corrado Poggesi, Iacopo Olivotto, Cecilia Ferrantini

**Affiliations:** ^1^Division of Physiology, Department of Experimental and Clinical Medicine, Università degli Studi di Firenze, Florence, Italy; ^2^Cardiomyopathy Unit, Careggi University Hospital, Florence, Italy; ^3^Department of NeuroFarBa, Università degli Studi di Firenze, Florence, Italy; ^4^LENS, Università degli Studi di Firenze and National Institute of Optics (INO-CNR), Florence, Italy; ^5^Metabolic Unit, A. Meyer Children’s Hospital, Florence, Italy; ^6^Pediatric Cardiology, Meyer Children’s Hospital, Florence, Italy

**Keywords:** dilated cardiomyopathy (DCM), duchenne muscular dystrophy (DMD), dystrophin (DMD), hiPSC-cardiomyocyte, stem cell models

## Abstract

Familial dilated cardiomyopathy (DCM) is mostly caused by mutations in genes encoding cytoskeletal and sarcomeric proteins. In the pediatric population, DCM is the predominant type of primitive myocardial disease. A severe form of DCM is associated with mutations in the *DMD* gene encoding dystrophin, which are the cause of Duchenne Muscular Dystrophy (DMD). DMD-associated cardiomyopathy is still poorly understood and orphan of a specific therapy. In the last 5 years, a rise of interest in disease models using human induced pluripotent stem cells (hiPSCs) has led to more than 50 original studies on DCM models. In this review paper, we provide a comprehensive overview on the advances in DMD cardiomyopathy disease modeling and highlight the most remarkable findings obtained from cardiomyocytes differentiated from hiPSCs of DMD patients. We will also describe how hiPSCs based studies have contributed to the identification of specific myocardial disease mechanisms that may be relevant in the pathogenesis of DCM, representing novel potential therapeutic targets.

## Highlights

• Mutations in the *DMD* gene (encoding dystrophin) account for 2% of inherited dilated cardiomyopathy (DCM). Advances in *in vitro* disease modeling using induced pluripotent stem cell-derived cardiomyocytes (hiPSC-CMs) may help develop specific therapies aimed at restoring or blunting myocardial damage and dysfunction in Duchenne and Becker Muscular Dystrophy.
• This review focuses on the physiological role of dystrophin in cardiomyocyte function during cardiac development and disease progression. The pathological mechanisms caused by the absence of dystrophin or the presence of truncated dystrophin isoforms are discussed. We provide an overview of the most recent and remarkable results obtained using hiPSC-CM lines with DMD mutations.
• Many studies employed hiPSC-CMs for pilot tests of gene therapy strategies. *In vitro* models such as DMD patient-cardiomyocytes offers a limitless source of human tissue to explore novel disease mechanisms that may represent targets for pharmacological intervention, tested and validated using novel high-throughput cell-screening techniques. Dystrophin-associated cardiomyopathy is an example of a rare cardiac disease where stem cell-based disease-modeling may help developing truly “patient-specific” therapeutic strategies.

Mutations in the dystrophin gene at the Xp21.1 locus are associated with devastating X-linked skeletal muscle disorders, such as Duchenne or Becker muscular dystrophies (DMD/BMD), and account for <2% of dilated cardiomyopathy (DCM) cases. The incidence of DMD is 1/5,000 male births (Mendell et al., 2012). Despite its low prevalence (<3/10,000), dystrophin-associated cardiomyopathy is the form of DCM that has been modeled *in vitro* more extensively using induced pluripotent stem cell-derived cardiomyocytes (iPSC-CMs) from patients. As a case in point, twenty original studies based on the use of DMD/BMD iPS-derived cell lines are present in the recent literature (2015–2019), as opposed to only few isolated examples for other genetically determined DCMs (Table 1). Why is dystrophin-associated cardiomyopathy more widely studied *in vitro* compared to other forms of DCM? And are DMD/BMD *in vitro* models specific in recapitulating dystrophin-related pathophysiology, or can lessons be learned regarding the wider spectrum of DCM?

**TABLE 1:** Principal pathogenic gene mutations described in genetic DCM along with their clinical and cellular phenotype.

Gene	Prevalence	Clinical phenotype	#hiPSC studies (References)	Functional output
Titin (*TTN*)	19–25% of familial forms 11–18% of sporadic forms	Usually milder forms of DCM, with LV reverse remodeling described after OMT. Can be associated with tibial muscle dystrophy and HCM (McNally and Mestroni, 2017). Truncating variants are related to alterations in mitochondrial function, increased interstitial fibrosis and reduced hypertrophy, along with increased ventricular arrhythmias at long-term follow-up, with a similar survival and overall cardiac function with respect to idiopathic DCM (Verdonschot et al., 2018).	5 (Hinson et al., 2015; Streckfuss-Bomeke et al., 2017; Chopra et al., 2018; Schick et al., 2018; Zaunbrecher et al., 2019)	Contractile deficit
Lamin A/C (*LMNA*)	5–6% of genetic DCM	Malignant DCM characterized by young onset, high penetrance, dysrhythmias (sinus node dysfunction, AF, atrioventricular node dysfunction, VT, VF, SCD), LV dysfunction and HF with reduced survival and frequent need for HT. Cardiac conduction system disease usually precedes the development of LV dilation and dysfunction (Hasselberg et al., 2018).	6 (Siu et al., 2012; Wyles et al., 2016a; Lee et al., 2017; Bertero et al., 2019; Salvarani et al., 2019; Shah et al., 2019)	LMNA haploinsufficieny; conduction defects; contractile defects
β-Myosin heavy chain (*MYH7*)	3–4% of DCM	Sarcomeric rare variant carriers show a more rapid progression toward death or HT compared to non-carriers, particularly after 50 years of age (Merlo et al., 2013).	?	
Cardiac troponin T (*TNNT2*)	3% of DCM	Clinical and prognostic profiles depend on type of mutation: carriers of Arg92Gln mutation have a worse prognosis than those with other mutations in *TNNT2* or other sarcomeric genes (Ripoll-Vera et al., 2016).	6 (Sun et al., 2012; Wu et al., 2015; Broughton et al., 2016; Burridge et al., 2016; Wang L. et al., 2018; Shafaattalab et al., 2019)	Calcium handling abnormalities; contractile defects
Type V voltage-gated cardiac Na channel (*SCN5A*)	2–3% of DCM	Arrhythmias (commonly AF) and myocyte dysfunction leading to progressive deterioration of LV systolic function (Bondue et al., 2018). Overlapping phenotypes: LQT, Brugada.	1 (Moreau et al., 2018)	Electrophysiological defects; Arrhythmias
RNA-binding protein 20 (*RBM20*)	1–5% of DCM	Malignant arrhythmic phenotype with high frequency of AF and progressive HF (Ripoll-Vera et al., 2016).	3 (Wyles et al., 2016a, b; Streckfuss-Bomeke et al., 2017)	Calcium handling abnormalities; contractile defects
Desmoplakin (*DSP*)	2% of DCM	Associated with Carvajal syndrome (autosomal recessive genetic disorder characterized by woolly hair, striate palmoplantar keratoderma and DCM). Additional phenotypic signs: dental abnormalities and leukonychia. LV dilatation usually asymptomatic at an early age. DCM progresses rapidly, leading to HF or SCD in adolescence (Yermakovich et al., 2018).	1 (Ng et al., 2019)	ACM
Dystrophin (*DMD*, Xp21.1 locus 16)	<2% of genetic DCM	Associated with Duchenne and Becker muscular dystrophy. Severe cardiac involvement in Duchenne (milder and later onset in Becker muscular dystrophy) with supraventricular arrhythmias, atrio-ventricular blocks and right bundle branch block, progressive LV dysfunction and HF (Mestroni et al., 2014).	20 (Dick et al., 2013; Guan et al., 2014; Zatti et al., 2014; Lin et al., 2015; Macadangdang et al., 2015; Japp et al., 2016; Nanni et al., 2016; Young et al., 2016; Kyrychenko et al., 2017; Zhang et al., 2017; Long et al., 2018; Caluori et al., 2019; Eisen et al., 2019; Farini et al., 2019; Jelinkova et al., 2019; Min et al., 2019; Pioner et al., 2019a; Sato et al., 2019; Tsurumi et al., 2019; Moretti et al., 2020)	Calcium handling abnormalities; contractile defects
α-Tropomyosin (*TPM1*)	1–2% of DCM	Overlapping phenotypes: LVNC, HCM (McNally and Mestroni, 2017)	1 (Takasaki et al., 2018)	Sarcomere defects
Desmin (*DES*)	1–2% of DCM (Taylor et al., 2007)	Malignant phenotype associated with desminopathies and myofibrillar myopathy. Can cause a spectrum of phenotypes from skeletal myopathy, mixed skeletal–cardiac disease (“desmin-related myopathy”), and cardiomyopathy (DCM as well as HCM or RCM). DCM is typically preceded by skeletal myopathy and can be associated with conduction defects (Mestroni et al., 2014).	1 (Tse et al., 2013)	DES protein aggregates
Filamin C (*FLNC*)	1% of DCM	Cardiomyopathy associated with myofibrillar myopathy and LVNC; high rate of ventricular arrhythmias and SCD (particularly in truncating variants) (Ader et al., 2019).	?	
Metavinculin (*VCL*)	1% of DCM	Can cause either DCM or HCM phenotype (Vasile et al., 2006)	?	
Phospholamban (PLN)	Rare (except for Netherlands where prevalence reaches 15% of DCM due to R14del mutation with founder effect) (van der Zwaag et al., 2012)	Early onset DCM with lethal ventricular arrhythmias. Low QRS complex potentials and decreased R wave amplitude, negative T waves in left precordial leads (Hof et al., 2019). PLN R14del mutation associated with high risk for malignant arrhythmias and end-stage HF from late adolescence, can cause either a DCM phenotype or ARVC (Mestroni et al., 2014). A milder phenotype is also reported (DeWitt et al., 2006).	4 (Karakikes et al., 2015; Stillitano et al., 2016; Ceholski et al., 2018; Stroik et al., 2019)	Electrophysiological defects
α-/β-/δ-Sarcoglycan (SGCA, SGCB, SGCD)	Rare	Recessive mutations in δ-sarcoglycan linked to limb girdle muscular dystrophy 2F, dominant mutations in δ-sarcoglycan linked to DCM (Campbell et al., 2016).	?	Ca handling abnormalities; Contractile defects
α-cardiac actin (*ACTC1*)	Rare	Familial atrial septal defect combined with a late-onset DCM (Frank et al., 2019). Can be associated with HCM and LVNC	?	
Cardiac troponin I (*TNNI3*)	Rare	Overlapping phenotype: HCM (McNally and Mestroni, 2017).	1 (Chang et al., 2018)	Telomere shortening
Cardiac troponin C (*TNNC1*)	Rare	Overlapping phenotypes: LVNC, HCM (McNally and Mestroni, 2017).	?	
Troponin I–interacting kinase (*TNNI3K*)	Rare	Conduction defect, AF (McNally and Mestroni, 2017).	?	
α-actinin 2 (ACTN2)	Rare	Overlapping phenotype: LVNC (McNally and Mestroni, 2017).	?	
BCL2-associated athanogene 3 (*BAG3*)	Rare	Associated with myofibrillar myopathy (McNally and Mestroni, 2017).	1 (Judge et al., 2017)	Disrupted myofibril; Contractile deficit
α-B-crystallin (*CRYAB*)	Rare	Associated with protein aggregation myopathy (McNally and Mestroni, 2017).	1 (Mitzelfelt et al., 2016)	Protein Aggregates; cellular stress
Titin-cap/telethonin (*TCAP*)	Rare	Associated with limb-girdle muscular dystrophy (McNally and Mestroni, 2017).	?	
Muscle LIM protein (*CSRP3*)	Rare	Overlapping phenotype: HCM (McNally and Mestroni, 2017).	1 (Li et al., 2019)	Calcium handling abnormalities
Cardiac ankyrin repeat protein (*ANKRD1*)	Rare	Associated with congenital heart disease (McNally and Mestroni, 2017).	?	
Cipher/ZASP (*LDB3*)	Rare	Overlapping phenotype: LVNC (McNally and Mestroni, 2017).	?	
Nebulette (*NEBL*)	Rare	Overlapping phenotypes: LVNC, HCM (McNally and Mestroni, 2017).	?	
Emerin (*EMD*)	Rare	Associated with Emery–Dreifuss muscular dystrophy (McNally and Mestroni, 2017).	1 (Shimojima et al., 2017)	Calcium handling abnormalities
Sulfonylurea receptor 2A, component of ATP-sensitive potassium channel (*ABCC9*)	Rare	Associated with AF, Osteochondrodysplasia (McNally and Mestroni, 2017).	?	
Potassium channel (*KCNQ1*)	Rare	Associated with AF, LQT1, short QT1, Jervell and Lange-Nielsen syndrome (McNally and Mestroni, 2017).	?	
HSP40 homolog, C19 (*DNAJC19*)	Rare	Associated with 3-methylglutaconic aciduria, type V (McNally and Mestroni, 2017).	1 (Rohani et al., 2020)	Mitochondrial abnormalities
Tafazzin (*TAZ/G4.5*)	Rare	Associated with LVNC, Barth syndrome, endocardial fibroelastosis 2 (McNally and Mestroni, 2017).	1 (Wang et al., 2014)	Mitochondrial defects; contractile defects

The first answer is simple: an intense and growing interest in DMD/BMD models derives from its potential for gene therapy. Given the severity of skeletal muscle damage associated with dystrophin depletion, a number of gene-targeted therapies aimed at inducing dystrophin expression have been developed. These promising gene-editing approaches have been developed for DMD to a level far beyond what has been achieved for any other cardiac or skeletal muscle diseases. Gene-editing strategies are being thoroughly evaluated *in vitro* in hopes of improving/delaying cardiac as well as muscular involvement in DMD patients.

The second issue is far more challenging and largely unresolved. Lack of dystrophin and of its stabilizing effects on the cytoskeleton causes a variety of downstream pathogenic mechanisms, ultimately leading to calcium dysregulation and sarcomere dysfunction. Whether and how all or part of these pathways are relevant to other forms of genetically-driven or acquired DCM, remains to be studied. In light of the advancements introduced by iPSC-derived cardiomyocyte modeling, the present review aims to describe the principal pathophysiological mechanisms associated with DMD, with the purpose of defining to what extent these mechanisms are specific to dystrophin depletion or rather, they are part of the wider spectrum of DCM-related cardiac abnormalities. Finally, we will discuss their implications for therapeutic discovery and tailored patient management.

## The Emerging Spectrum of Genetic DCM

DCM is a disease of the myocardium characterized by left ventricular (LV) dilatation and dysfunction with an estimated prevalence of 1:2,500, an incidence of 7:100,000 and a male to female ratio of 3:1, in adults (Rakar et al., 1997). Recent estimates suggest a considerably higher prevalence of ≥1 in 250 individuals (Hershberger et al., 2013). In the pediatric population, DCM is the predominant type of cardiomyopathy and its incidence is higher in the first year of life (Lipshultz et al., 2003). DCM is one of the leading causes of heart failure (HF) and the most frequent indication for heart transplantation (Japp et al., 2016).

In almost 40% of cases, DCM is genetically determined and associated with mutations in genes coding for titin (19–25%), lamin (5–6%), and sarcomeric thin (1–3%) or thick (3–4%) filament proteins. A variety of pathways and cellular structures are affected by these mutations with negative effects on mechanisms like calcium homeostasis, generation–transmission of mechanic force in the myocardium, muscle contraction and ion channel function. The prevailing pattern of inheritance is autosomal dominant, with incomplete, penetrance, and variable age-related expression. The complex interaction between common and rare variants, the presence of additional modifier gene mutations or polymorphisms, as well as a number of environmental factors, may be responsible for the clinical heterogeneity of DCM even in families carrying the same pathogenic gene variant (Bondue et al., 2018). While the etiology of a wide proportion of cases remains unknown after genetic testing (the so-called “idiopathic” DCM), these apparently “non-genetic” forms, even when clearly acquired (e.g., alcohol- or chemotherapy-related or post-partum cardiomyopathies), are often influenced by the individual genetic profile (McNally and Mestroni, 2017). On the other hand, the pathogenicity of a gene mutation is modulated by interfering factors like age, the hormonal milieu, the status if the innate immune system, the presence of comorbidities such as chronic inflammatory diseases or hypertension, inherited mitochondrial alterations or the exposure to specific environmental triggers (such as cardio-toxic substances or drugs); all these can profoundly alter the clinical phenotype as well as the outcome. In this complex scenario, understanding genotype–phenotype correlations represents the ultimate challenge for translational research in the field of cardiomyopathies.

Mutations in the *TTN* gene coding for titin, a giant protein that works as a nano-spring, account for most known genetic forms of DCM (19–25% of familial forms and 11–18% of sporadic forms), with incomplete penetrance and variable expression, frequently (but not always) presenting with relatively mild phenotypes and slow progression (Bondue et al., 2018). Conversely, mutations in the *LMNA* gene (lamin A/C, representing 5–6% of genetic DCM) (Hasselberg et al., 2018), *FLNC* (filamin C) (Ortiz-Genga et al., 2016), *DES* (desmin) (Capetanaki et al., 2015), *PLN* (phospholamban) (Schmitt et al., 2003), *SCN5A* (McNair et al., 2011), and mutations of *RBM20*, the regulator of *TTN* splicing (Refaat et al., 2012), have been identified as malignant causes of DCM, featuring a marked arrhythmic propensity in patients. The electrical instability characterizing these forms gives rise to a clinical overlap between DCM, arrhythmogenic cardiomyopathy and channelopathies (Table 1), as these different disease may share a similar cellular and molecular basis of arrhythmogenesis, ultimately leading to an increased risk of sudden cardiac death (Gigli et al., 2019). Currently, lamin A/C gene mutations represent the only instance where the presence of a specific genetic background *per se* modifies clinical management, warranting consideration for the implantation of cardioverter defibrillators (ICDs), regardless of the severity of LV dysfunction (Priori et al., 2015).

## Clinical Profile of Dystrophin-Associated DCM

**FIGURE 1:**
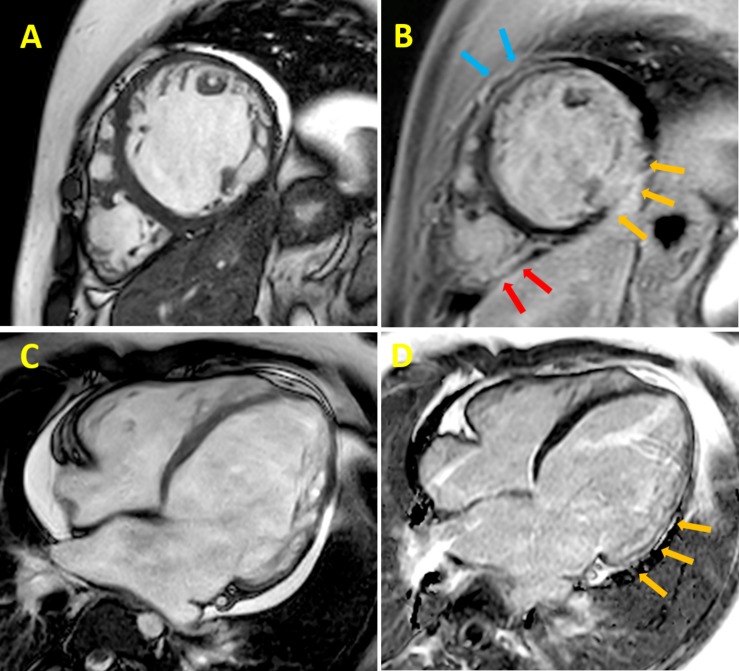
Cardiac magnetic resonance (CMR) cine imaging of a 24 years old DMD patient. The collection of representative images from patients was approved by the Ethical Committee of the Meyer Pediatric Hospital of Florence in the context of a project funded by Telethon Italy (grant GGP16191). Informed consent to patients was performed conform the declaration of Helsinki. This data does not contribute to any novel finding. **(A)** Late gadolinium-enhancement **(B)** left ventricular (LV) short-axis section images of a patient with Duchenne muscular dystrophy (DMD). Yellow arrows indicate the inferolateral subepicardial and midwall contiguous fibrosis; light blue arrows indicate the anterior segment and the red arrows the posterolateral right ventricle wall, both showing midwall fibrosis; CMR cine imaging **(C)** and late gadolinium-enhancement **(D)** LV long-axis section images of the same patient with DMD; yellow arrows indicate midwall fibrosis of the inferolateral segment.

DMD is a severe disorder diagnosed in childhood: without supportive care, young men with DMD typically die in their late teens or early 20 s. Historically, the most common cause of death has been respiratory failure. However, with improved respiratory support and the introduction of steroid therapy, DMD cardiomyopathy is becoming an increasingly important cause of morbidity and mortality, due to HF and ventricular arrhythmias leading to sudden death (Eagle et al., 2002; McNally, 2007). From the clinical perspective, dystrophin-associated cardiomyopathy is characterized by a consistently severe phenotype (Table 1), and progresses relentlessly. Regional progression of contractile impairment (from the inferolateral wall to the rest of the LV) and diffuse/transmural replacement fibrosis of the inferolateral LV wall are common features of this form of DCM, relatively specific instrumental markers that help distinguish DMD-cardiomyopathy from other forms of DCM (Frankel and Rosser, 1976). LV dimensions and myocardial stiffness increase progressively as the extension of myocardial fibrosis (MF) increases (Figure 1). This, in turn, leads to an increased cardiac workload and to the activation of the renin angiotensin and the sympathetic nervous systems, thus leading to worse cardiac function and more severe HF-related symptoms and signs (Perloff, 1984). DMD patients are usually extremely limited in their mobility since the first decade, so that HF-related symptoms are often blunted by the relative lack of physical activity: overt cardiac symptoms have been reported in less than 30% of patients aged <18 (Nigro et al., 1990), underestimating the true extent and prevalence of HF in DMD subjects. Patients may complain about palpitations, dizziness or fainting, the latter commonly associated with the presence of conduction abnormalities at ECG (Fayssoil et al., 2017). Dyspnea is controlled by the mechanical ventilation used for neuromuscular respiratory failure: in this setting, peripheral edema and ascites are common findings and are related to the ventilator-driven positive intrathoracic pressures that reduce venous return (Nigro et al., 1990). Pleural effusion is frequently seen in end-stage disease (Fayssoil et al., 2014). Once HF appears the prognosis is poor, despite the extensive use of the complete therapeutic armamentarium including neuro-hormonal blockade. This is in contrast with other genetic forms of DCM where therapy can alter the progression of LV dysfunction and reverse remodeling is common (Japp et al., 2016; Ader et al., 2019). So far, no clear association between specific dystrophin genotypes and the severity of the cardiac phenotype has been established.

ECG abnormalities are often seen in DMD patients: the most frequently reported changes are sinus tachycardia, short PR intervals, deep and narrow “pseudo-necrosis” Q waves in inferolateral leads, tall R wave in the right precordial leads, right bundle branch block and flat/inverted T waves (Manning and Cropp, 1958; Takami et al., 2008). Arrhythmias such as atrial flutter (Perloff, 1984), non-sustained or sustained ventricular tachycardia (Corrado et al., 2002), complete atrioventricular blocks and sick sinus syndrome (Fayssoil et al., 2008, 2010) are seen mainly in patients with severe LV dysfunction (Gigli et al., 2019). Doppler echocardiography typically shows early LV dilatation and dysfunction, beginning in the pediatric age range (McNally et al., 2015). Abnormal indices of diastolic function have been proposed as early indicators of myocardial abnormalities, preceding the development of chamber enlargement and systolic dysfunction in the early stages of DMD-related DCM (Markham et al., 2006). Likewise, regional myocardial abnormalities have been reported in early stages, affecting mainly the inferolateral region of the LV (Giatrakos et al., 2006). Mitral functional regurgitation may also be present, as a consequence of LV and mitral annulus dilation. In the recent years, cardiac magnetic resonance (CMR) has been demonstrated to be a valuable tool for detection of early cardiac involvement and prediction of ventricular arrhythmias, cardiac remodeling and long-term outcome (Mertens et al., 2008; McNally, 2017; Silva et al., 2017).

Management of DMD cardiomyopathy is challenging, due to the interplay of respiratory and cardiac manifestations, poor response to standard treatment and severe systemic manifestations of DMD. Moreover, large-scale randomized clinical trials (RCTs) are rare in this field. Therefore, the latest guidelines for the management of DMD cardiomyopathy are mostly based on the consensus of experts from a wide range of disciplines (Rakar et al., 1997; McNally et al., 2015). Specifically, in the absence of dystrophin-specific targeted cardiac therapies, the 2014 NHLBI working group recommended the introduction of angiotensin-converting enzyme inhibitors (ACEi) or angiotensin receptor blockers (ARBs) in boys with DMD by 10 years of age, in addition to traditional HF treatments (i.e., beta-blockers, mineralocorticoid receptor antagonists, diuretics), as LV dysfunction progresses (Silva et al., 2017). Steroid therapy primarily used to prolong ambulation, also showed a cardioprotective effect in DMD patients (Markham et al., 2008). Early initiation of noninvasive nocturnal ventilation increases long-term survival (McNally and Mestroni, 2017). The surveillance and management of arrhythmias in DMD patients is currently based on general adult HF recommendations. However, aggressive options such as an implantable cardioverter defibrillator (ICD) or the use of cardiac resynchronization therapy (CRT) is controversial in these patients, due to the limited life expectancy and the increased procedural risk (Wang M. et al., 2018). The same considerations apply to mechanical circulatory support (Iodice et al., 2015) and left ventricular assist devices (LVAD), rarely appropriate in this setting. Cardiac transplant is generally contraindicated in DMD (Rakar et al., 1997).

## Dystrophin and Dystrophin-Glycogen Complex

Dystrophin (DMD gene), the largest known gene in the human genome (2.5 million base pairs) (Mandel, 1989), was also the first found to be associated with DCM (Towbin et al., 1993). Ninety-nine percent of the mRNA transcribed from DMD gene is made by introns (non-coding sequences) and the coding sequence accounts for 86 exons. The full-length mRNA (14,000 bp) has a low tissue specificity and is detectable almost ubiquitously. Conversely, protein expression is confined to striated muscle and the neuropil (areas of the nervous system composed of mostly unmyelinated axons, dendrites and glial cell processes that forms a synaptically dense region containing a relatively low number of cell bodies). The obvious discrepancy between mRNA and protein distribution remains unresolved (Vogel and Marcotte, 2012). High levels of full-length dystrophin protein are present in both cardiac and skeletal myocytes, while the protein has a lower expression in unmyelinated axons, dendrites and glial cell processes forming synapsis-dense regions of the brain (“neuropil”). Notably, the DMD gene produces many protein isoforms for different tissue-specific functions (Muntoni et al., 2003), owing to alternative splicing events. Splice variants are formed by *exon skipping*, i.e., exclusion of exons from primary transcripts, or by *exon scrambling*, i.e., subversion of the reciprocal order of exons in the transcript (Muntoni, 2003).

**FIGURE 2:**
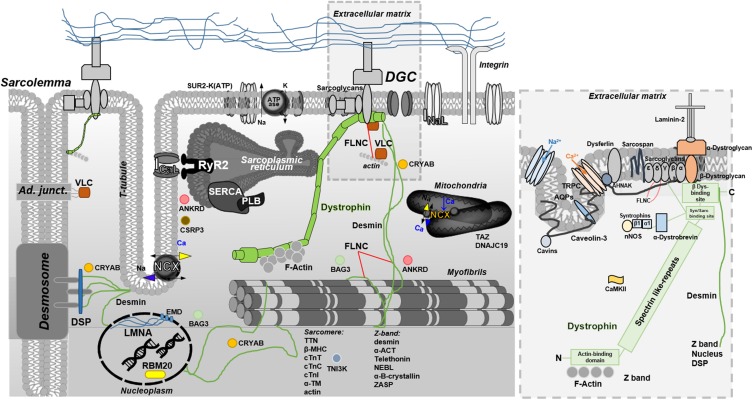
Overview of full-length dystrophin in the context of other DCM-related proteins. The blow-up box, is a focus on full-length dystrophin structure and interactions. Full-length dystrophin is a large rod-shaped protein with a molecular weight of 427 kDa composed by 4 structural domains. The amino (N)-terminal domain has homology with α-actinin and binds, in particular, the F-actin; the central rod-domain contains 24–25 spectrin-like repeats the cysteine-rich domain intereacting with syntrophin and sarcoglycans; the last carboxy (C)-terminal domain associates at the C-terminal with β-dystroglican and several other proteins to form a major protein complex referred to as the dystrophin glycoproteic complex (DGC) (Hoffman et al., 1987; Ervasti and Campbell, 1993). The DGC consists of α- and β-dystroglycan subunits, α-, β-, δ-, γ-, and ε-sarcoglycans, sarcospan, α- and β-syntrophins, α-dystrobrevin, and neuronal nitric oxide synthase (nNOS) (Mosqueira et al., 2013). DGC related-pathways include Ca^2+^ homeostasis and E-C coupling, mitochondrial function, motor protein interaction (sarcomere/Z-band), and gene expression. For instance, the acetylcholine receptor, the skeletal and cardiac isoforms of the voltage-gated sodium channels (Nav1.4 and Nav1.5, respectively), the L-type Ca^2+^ channel, aquaporin, and stretch-activated channel or transient receptor potential (TRP) cation channels (Shirokova and Niggli, 2013) are closely associated with the DGC via syntrophins. In the cardiac tissue, dystrophin is also associated to: Cavin-1 and Caveolin-3 (responsible for caveolae/T tubule formation), Ahnak1 (modulates L-type Ca^2+^ channel), CryAB (involved in cytoprotection and antiapoptosis), and Cipher (plays a role in muscle contraction maintaining the Z-line integrity and signaling). Dystrophin can be also target of phosphorylation by Calmodulin-dependent kinase II (CaMKII) that modulates the affinity for F-actin and syntrophin (Madhavan and Jarrett, 1994). Other short isoforms of dystrophin come from spliced variants and are expressed in several other tissues. In particular, the Dp71 is expressed in cardiac muscle and likely present in T-tubular membranes (Kaprielian and Severs, 2000; Kaprielian et al., 2000).

Full-length dystrophin (depicted in Figure 2, in the context of other proteins associated with DCM) is a large rod-shaped protein with a molecular weight of 427 kDa, composed by 4 structural domains. The amino (N)-terminal domain has homology with α-actinin and binds, among other proteins, F-actin. The central rod-domain contains 24–25 spectrin-like repeats. The third one is a cysteine-rich domain. The last carboxy (C)-terminal domain associates at the C-terminal with several other proteins to form a major protein complex referred to as the dystrophin-glycoproteic complex (DGC) (Hoffman et al., 1987; Ervasti and Campbell, 1993). The DGC consists of α- and β-dystroglycan subunits, α-, β-, δ-, γ-, and ε-sarcoglycans, sarcospan, α- and β-syntrophins, α-dystrobrevin, and neuronal nitric oxide synthase (nNOS) (Mosqueira et al., 2013). A central role of dystrophin within this network is evidenced by the collapse of the entire DGC in the absence of dystrophin (Leyva-Leyva et al., 2018). Dystrophin is localized at the cytoplasmic face of the muscle cell plasma membrane, or sarcolemma, particularly within a cytoskeletal lattice termed costameres. Through an extensive network of interacting proteins (Ervasti, 2003) costameres physically couple the sarcolemma with the Z disk of force-generating myofibrils (Rahimov and Kunkel, 2013). In the striated muscle, the dystrophin network covers almost the entire cytoplasmic surface of the plasma membrane. It is strategically placed to serve in as a shock absorber, promoting membrane stability and transduction of mechanical force from the extracellular matrix during muscle contraction/stretch. A single-molecule analysis by atomic force microscopy revealed that dystrophin, by stretching with stochastic unfolding and refolding of its central domain, is able to keep elastic forces around 25 pN over a significant length change up to ∼800 nm (90% of fully unfolded central domain length), an extension range that is close to the change of sarcomere length during myofilament contraction in striated muscle (Le et al., 2018). In addition, the DGC serves a variety of signaling pathways that regulate several myocyte functions including Ca^2+^ homeostasis and excitation-contraction coupling, mitochondrial function, motor protein interaction, and gene expression. For instance, the acetylcholine receptor, the skeletal and cardiac isoforms of the voltage-gated sodium channels (Nav1.4 and Nav1.5, respectively), the L-type Ca^2+^ channel, aquaporin, stretch-activated channels and other transient receptor potential (TRP) cation channels (Shirokova and Niggli, 2013) are closely associated with the DGC via syntrophins and are likely modulated by changes of dystrophin conformation. Moreover, in the myocardium dystrophin appears to serve as a signaling scaffold and membrane protein organizer, targeted by numerous signaling molecules that participate in a diverse set of pathways. For instance, it is also associated with Cavin-1 and Caveolin-3 (responsible for caveolae/T tubule formation), Ahnak1 (modulator of L-type Ca^2+^ channel), CryAB (involved in cytoprotection and antiapoptosis), and Cipher (involved in maintaining Z-line integrity and signaling). Dystrophin can be a target of phosphorylation by Calmodulin-dependent kinase II (CaMKII), modulating the affinity for F-actin and syntrophin (Madhavan and Jarrett, 1994). Other short isoforms of dystrophin originate from spliced variants and are expressed elsewhere. Specifically, Dp71 is expressed in cardiac muscle and is likely present in the T-tubular membrane (Kaprielian and Severs, 2000; Kaprielian et al., 2000). Another important protein is utrophin, a large (376 kDa) cytoskeletal protein, autosomal paralog of dystrophin with similar binding partners and structure that have been used to replace dystrophin in striated muscle, as a therapeutic strategy for DMD (Blake et al., 1996). Dystrophin and utrophin exhibit marked differences in amino acid sequence, particularly within the large rod domain. Thus, it is possible that proteins with unique specificity for dystrophin or utrophin exist, though specific binding properties remain to be identified.

Mutations in DMD that shift the reading frame generate a truncated protein, which cannot anchor itself to the glycoprotein complex. The protein is, therefore, unstable and present at undetectable levels in muscle tissue. It is widely accepted that the predominant functional consequence of the lack of dystrophin is cellular vulnerability to mechanical stress associated with muscle contraction. Membrane fragility and altered Ca^2+^ homeostasis stem from the loss of dystrophin in both skeletal muscle fibers and cardiomyocytes, as demonstrated by studies in DMD animal models. These studies are consistent in showing that the absence of dystrophin leads to altered myogenesis and myofiber commitment in skeletal muscle, originally observed in X Chromosome-Linked Muscular Dystrophy (*mdx*, genetic mouse model of DMD that completely lacks full-length dystrophin) mouse embryos (Merrick et al., 2009) and skeletal muscle biopsies from human fetuses affected by DMD (Farini et al., 2016). Myogenesis was severely disrupted in mdx embryos, which displayed developmental delays including altered myotube size and shape, lower number of myotubes, displacement defects and aberrant Pax7-positive skeletal muscle stem cell behavior (Merrick et al., 2009). Moreover, Dumont and collaborators demonstrated that dystrophin is highly expressed in satellite cells (skeletal muscle stem cells) and regulates their polarity and asymmetric division. Hence, loss of dystrophin results not only in myofiber fragility but also – due to strikingly reduced asymmetric divisions – in the lack of regenerating myofibers (Dumont et al., 2015). Farini and collaborators proposed that PLC/IP3/IP3R/RyR1/Ca^2+^ signaling pathway may be activated differently in fetal compared to adult DMD skeletal muscle fibers, and proposed that this mechanism was directly linked to defects in myofiber formation and to a rearrangement in the expression of fast/slow-muscle specific genes (Farini et al., 2016).

### A Developmental Role for Dystrophin in the Heart

The role of dystrophin during human cardiac development is still not completely deciphered. The absence of a shock absorber that may preserve membrane homeostasis and may be involved in several intracellular pathways is supposed to negatively affect physiological human myocardium formation. Previous studies reported that dystrophin is already expressed on the surface of cardiomyocytes in 8-week-old fetuses (Chevron et al., 1994) and, while no studies have investigated earlier phases of gestation, some authors hypothesize that specific fetal dystrophin isoforms may be present during early embryo development (Mora et al., 1994). Of note, a greater quantity of dystrophin was found in cardiac than skeletal muscle in fetuses, likely related to the earlier development of the myocardium. In the same study, Mora and colleagues reported that DGC proteins were expressed variably in period between 8 and 12 weeks of gestation. The expression of spectrin, a DGC-protein involved in the maintenance of plasma membrane integrity and cytoskeletal structure, was detected earlier than vinculin and talin (cytoskeletal proteins connecting integrins), suggesting that dystrophin is probably required on the sarcolemma before these two proteins. At 8 weeks, β-dystroglycan were found patchily on cardiac cells and immunostaining intensity did not appear to change with heart maturation. Adhalin (α-Sarcoglycan) did not appear in the cardiac tissue at 8 weeks and showed limited expression at 12 and at 16 weeks. The dystrophin-like protein utrophin was found at 8 weeks and faintly increased from 8 to 16 weeks (Mora et al., 1996). Finally, other authors identified a distinct pool of dystrophin molecules associated with myofibrils and localized within the Z-disks of the sarcomeres in association with α-actinin and desmin, in cardiac but not in skeletal muscle (Meng et al., 1996). Whether contractile function and dystrophin distribution influence each other in the early stages of development is still unknown.

## Human Induced Pluripotent Stem Cell Derived Cardiomyocytes as a Model of Dystrophin-Associated Cardiomyopathy

The advent of human induced pluripotent stem cells (hiPSCs) has opened the door to the exploration of core biomedical questions using personalized, renewable patient-specific human cell models instead of animal models. Cardiomyocytes derived from hiPSCs represent a unique platform to penetrate the pathophysiological processes related to early-stage defects underlying the onset and progression of DMD cardiomyopathy and delineate the so-called “cellular” phenotypes. Recent validation of these cellular models compared to native myocardial tissue (Ronaldson-Bouchard et al., 2018), and the possibility to increase the level of maturation of hiPSCs using long-term cultures combined with nanostructured surfaces that mimic the extracellular matrix (Pioner et al., 2019a), will allow future research to comprehensively perform complex biophysical studies to simultaneously monitor membrane fragility, intracellular calcium handling and myofilament function. This represents a unique opportunity in the context of DCM, a disease rarely requiring surgical strategies and therefore difficult to study *in vitro* on human myocardial samples.

**FIGURE 3:**
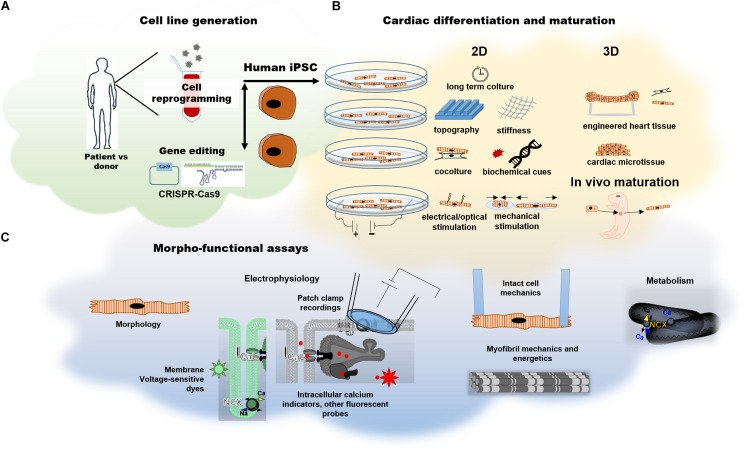
Human induced pluripotent stem cell derived cardiomyocytes. **(A)** Generation of hiPSCs by cell reprogramming of somatic cells from patients or healthy donors or via CRISPR-Cas9 gene editing for the generation of isogenic pairs. **(B)** Current maturation strategies for hiPSC-CMs at cell level (2D strategies) or in multicellular model (3D strategies). **(C)** Possible methods for the assessment of electrophysiological and contractile properties.

Notably, studies using patient-derived tissues often represent the final stages of disease, therefore separating primary mechanisms (i.e., those induced by the lack of full-length dystrophin) from the secondary mechanisms (i.e., those induced by chronic neurohormonal stimulation, adverse remodeling and effects of medications) is nearly impossible. On the other hand, small rodent models recapitulate facets of the human cardiomyopathy phenotype at the early stages of disease development. However, rat and mice cardiomyocytes differ from human cells in the expression of key contractile proteins, heart rate, electrical properties and ion channel function, often making it difficult to translate findings to humans. Cardiomyocytes obtained from the differentiation of iPSCs are the most prominent model to date for modeling human heart *in vitro*. Since the first report of Yamanaka and Takahashi in 2006, the reprogramming of somatic cells into embryonic-like induced pluripotent stem cells (iPSC) has becoming a widely used strategy for disease-in-a-dish modeling (Takahashi and Yamanaka, 2006). Generation of iPSCs bypass ethical concerns regarding disruption of embryos and, differently from embryonic stem cells (ESCs), can be derived directly from patients or healthy donors. Patient-derived iPSCs (hiPSCs) today provide the opportunity to characterize specific disease phenotypes in different tissues, thanks to tissue-specific differentiation protocols. Furthermore, targeting specific genes by CRISPR-Cas9 gene editing technology is a major resource allowing phenotyping of any cardiomyopathy-associated mutation, as well as a direct testing of the pathogenicity of variants of uncertain significance (Figure 3A).

It must be emphasized that, even after differentiation into cardiomyocytes, hiPSC-CMs retain immature features resembling developmental cardiomyocytes. In the very early phases (day 20–30 post differentiation), hiPSC-CMs exhibit fetal-type proteomic patterns (van den Berg et al., 2015), automaticity (spontaneous contraction), fetal-like ion channel expression (Davis et al., 2011), contractile properties (Pioner et al., 2016), and metabolism (Ulmer and Eschenhagen, 2019). A developmental-like behavior underlies the changes in hiPSC-CM structure and function. Using conventional culture conditions, cell growth, the organization of subcellular structures and the improvement of cardiomyocyte function are all time-dependent events. For instance, increase of repolarizing ion currents (I_*k*__1_ in particular) and reduction of the funny current (I_*f*_) decrease automaticity, together with a prolongation of cardiomyocyte action potential (Sartiani et al., 2007). Furthermore, sarcoplasmic reticulum calcium content becomes more prominent in the regulation of calcium handling (Itzhaki et al., 2006). Limited information is still available for the contractile apparatus. Sarcomere protein composition and isoform expression can be extremely variable in hiPSC-CMs. In a time window of 100 days after differentiation, hiPSC-CMs undergo sarcomere formation and isoform switch. This is similar to the process of fetal cardiac development (Sasse et al., 1993; Reiser et al., 2001; Racca et al., 2016), which occurs *in vitro* in a time- dependent manner. Furthermore, our current knowledge of sarcomere protein isoforms in hiPSC-CMs is limited by the lack of any truly effective chamber-specific differentiation protocol: for this reason, observations refer to the general cardiomyocyte population. At thick filament level, the cardiac fast alpha myosin heavy chain isoform (α-MHC, MHY6) and the atrial myosin regulatory light chain (MLC-2a, MYL7) are overall prevalent in hiPSC-CMs but long-term culture and stiff substrates (Weber et al., 2016) drive the isoform switch toward a predominant expression of the slow beta myosin heavy chain isoform (β-MHC, *MHY7*) and of the ventricular regulatory light chain (MLC-2v, *MYL2*) (Cai et al., 2019), and increase myosin binding protein C (cMyBP-C) expression (Cai et al., 2019). Titin (TTN) isoforms N2B and N2BA are co-expressed with a pattern similar to that found in the heart tissue of healthy individuals (Streckfuss-Bomeke et al., 2017). Sarcomeric protein composition is of extreme importance when modeling hiPSC-CM for the impact on contraction kinetics. In addition, some studies that used CRISPR-mediated genomic deletion in WT-hiPSC-CM lines for studying *de novo* sarcomere assembly reported that β-MHC, its connection with titin (Chopra et al., 2018) and the expression of the newly discovered Cronos titin (Zaunbrecher et al., 2019) are necessary for sarcomere assembly and function; non-muscle myosin II (NMM II) or α-MHC, instead, are not required. Another fundamental sarcomeric property is the Ca^2+^ buffering capacity of troponin C, which contributes to determine the Ca^2+^ sensitivity (pCa50) of myofibril tension development. Differences in thin filament protein isoform expression or phosphorylation patterns may account for changes in myofilament Ca^2+^ buffering. In hiPSC-CMs, prevalence of slow skeletal troponin I (ssTnI, TNNI1) may recapitulate properties typical of the embryonic age characterized by higher myofilament Ca^2+^ sensitivity and lower pH sensitivity, which may be particularly important for fetal hearts, which have modest amounts of Ca^2+^ delivered to the sarcomeres and are exposed to hypoxic conditions. As previously reported, the mechanisms responsible for the inhibition of myofilament force generation by acidosis include: (1) reduction in the affinity of troponin C (TnC) for Ca^2+^, (2) alterations in thin filament activation, especially the TnI-TnC interaction, and (3) inhibition of the actin-myosin interaction (Wolska et al., 2001). Long-term culture (>90 days after differentiation) have shown co-expression (almost 1:1) of ssTnI with the adult cardiac troponin I isoform (cTnI) (Bedada et al., 2014; Pioner et al., 2016). Similarly, co-expression of slow skeletal Troponin T (ssTnT) and cardiac troponin T (cTnT) has been reported (Iorga et al., 2017). Of note, using the current differentiation protocols, we and other groups have found myofibril force comparable to fetal myofibrils and kinetic properties that more closely reflect those of adult ventricular myofibrils expressing β MHC (Pioner et al., 2016; Racca et al., 2016; Iorga et al., 2017).

For this reason, maturation strategies applied to hiPSC-CMs are a primary challenge in the field. In the last few years, many researchers have explored ways to induce greater structure and function development of hiPSC-CMs, using a wide range of methods such as long-term culture (Lundy et al., 2013), biomimetic substrates (Macadangdang et al., 2014, 2015; Ribeiro et al., 2015), electrical or mechanical stimulation (Nunes et al., 2013), co-culture with other cell types (Tulloch et al., 2011), alteration of growth media (Birket et al., 2015) and 3D culture system such as engineered heart tissue (EHTs), heart on a chip or even *in vivo* neonatal rat heart system (Cho et al., 2017; Figure 3B). Since hiPSC-CMs possess features of developing cardiomyocytes, they can serve as human models for investigating the developmental time-course of early-stage cardiac diseases at cardiac cell level (Figure 3C).

### Modeling Early-Stages of DMD- Associated DCM Using hiPSC-CMs

Cardiomyocytes derived from patient-hiPSCs can be considered a unique platform to address early-stage defects underlying DMD cardiomyopathy in a developing human heart. Models for inherited DCM have been obtained for mutated genes involving sarcomere and sarcomere Z-band stability, E-C coupling proteins, cytoskeleton, mitochondria and desmosomes (Table 1). Models for relatively common (arrhytmogenic right ventricular cardiomyopathies, laminopathies) as well as rare genetic syndromes associated with DCM (Friedreich’s ataxia, Barth syndrome, carnitine palmitoyltransferase II deficiency, LEOPARD syndrome, Pompe disease) have been reported (Burridge et al., 2016). Many studies using hiPSCs sought to mirror the pathomechanisms found in end-stage human samples or animal models, although none addressed in-depth the potential for novel therapeutic targets. Patient-derived hiPSC-cardiomyocytes and genome editing of DMD via CRISPR-Cas9 or TALEN can provide direct evidence that the lack of dystrophin directly impacts cardiomyocyte function. In normal hiPSC-CMs, full-length dystrophin (Dp427) is expressed early after differentiation. Recently, mass spectroscopy analysis has suggested that dystrophin is upregulated in a period of time from days 30 to 60 post differentiation. This is also supported by similar results in the developmental expression pattern of embryonic and postnatal mouse hearts (Cai et al., 2019). A lack of information limits the comprehension of the physiological role and expression levels of the Dp71 isoform and of utrophin. However, a novel transient dystrophin isoform (Dp412 kDa) was identified in hiPSCs during mesodermal differentiation induced by a 72 h exposure to bone morphogenetic protein 4 (BMP4), suggesting a role of shorter isoforms during development (Massourides et al., 2015). Furthermore, Jelinkova et al. (2019) tested a WT hiPSC and two WT hESC lines and reported that Dp427 is already expressed before differentiation and that the absence of dystrophin leads to increased ROS production, NOS activity and DNA damage in DMD-hiPSCs (2 independent patients). These results further support the value of the hiPSC platform for modeling early-stages of DMD.

In such perspective, we have summarized the major findings and limitations of published DMD-hiPSC-CMs models in Table 2.

**TABLE 2:** DMD-cardiomyopathy studies based on hiPSC-CMs: individual mutations vs. functional parameter and therapeutic attempts *in vitro*.

Mutation/parameter	Membrane	Electrophysiology	Calcium handling	Sarcomere	Metabolism and oxidative stress	Therapeutic approach	References
Δ Exon 50	Fragility/damage		Slower calcium transients	↓ myofibril force, slower myofibril relaxation, ↑ myofibril calcium sensitivity	↑ mPTP opening; unaffected mitochondrial respiration		Guan et al., 2014; Macadangdang et al., 2015; Pioner et al., 2019a
Δ Exons 49–50	Fragility/damage	↑Spontaneous electrical activity	↑Intracellular diastolic calcium level	↑cTnI release (marker of cell damage)		ONX-0914 reduced ROS level	Farini et al., 2019
DMD; nonspecified mut.					Reduced Nup153 factor (regulates cardiac remodeling)		Nanni et al., 2016
Δ Exons 45–52	Fragility/damage		↑Intracellular diastolic calcium level	↓Sarcomere transcriptome	Mitochondrial damage, CASP3 activation, apoptosis	Poloxamer 188, reduced resting cytosolic Ca^2+^ level, CASP3 activation and apoptosis	Lin et al., 2015
c.263ΔG	Fragility/damage		Slower calcium transients	↓Alignment; ↓acto-myosin turnover; cellular hypertrophy			Macadangdang et al., 2015; Pioner et al., 2019a
Δ Exons 52–54					NOS-induced ROS release		Jelinkova et al., 2019
Δ Exons 43–45			↑Stretch-induced intracellular calcium entry				Tsurumi et al., 2019
Δ Exons 8–12		↑I_*Ca–L*_ density; prolonged APD					Eisen et al., 2019
c.5899C > T		↑I_*Ca–L*_ density; prolonged APD					Eisen et al., 2019
Δ Exon 8-9		↑Spontaneous electrical activity	Slower calcium transients	↓Force production		Rescue by CRISPR-Cas9-deletion of 3–9, 6–9, 7–11	Kyrychenko et al., 2017
Δ Exon 3–6					Mitochondrial damage; ↑ROS level ↑exosome protection	Exosome protection	Gartz et al., 2018
Δ Exons 45–50		↑Spontaneous electrical activity					Caluori et al., 2019
Δ Exons 48–50						CRISPR-Cas9 deletion of exons 45–55 restored DGC	Young et al., 2016
Δ Exons 46–55	Fragility/damage		Cellular arrhythmias			Exon 45 skipping with PMO improved arrhythmias	Sato et al., 2019
Δ Exon 44						CRISPR-Cas9 restoration	Min et al., 2019
Exon 45, 51, 45, 53, 44, 46, 52, 50, 43, 6, 7, 8, 55						CRISPR-Cas9 restoration	Long et al., 2018
Δ Exons 48–50				↓Force production		CRISPR-Cpf1 reframing of Exon 51 or exon skipping: restored dystrophin; enhanced contractile function.	Zhang et al., 2017
Δ Exons 4–43				↓Force production		Restoration by HAC carrying the full-length genomic dystrophin sequence	Zatti et al., 2014
Δ Exons 48–50, 47–50, Δ TG from exon 35, c.3217G > T						Antisense oligonucleotide-mediated skipping of exon 51 and delivery of dystrophin minigene	Dick et al., 2013
Δ Exon 52			Slower calcium transients; arrhythmic events			AAV6-Cas9-g51-mediated excision of exon 51 restored dystrophin expression and ameliorate skeletal myotube formation as well as abnormal cardiomyocyte Ca^2+^ handling and arrhythmogenic susceptibility	Moretti et al., 2020

### Excitation Contraction Coupling of DMD-hiPSC Cardiomyocyte Models

Calcium handling abnormalities have been described as a major consequence of dystrophin and other DCM-related mutations but the mechanistic progression remains unclear. A study from the Wu laboratory explored DCM-patient derived cell lines carrying the R173W-cTnT mutation, reporting reduced sarcoplasmic reticulum calcium content and higher rate of spontaneous arrhythmic electrical activity compared to normal cardiomyocytes (Sun et al., 2012). In this mutant cell line, decreased cell contractility was determined by atomic force microscopy and compared with lines obtained from the non-affected members of the same family. Overexpression of the sarcoplasmic reticulum Ca^2+^ adenosine triphosphatase (Serca2a) enhanced the intracellular Ca^2+^ transient and restored force production (Sun et al., 2012). These and other works have provided reports of stem cell lines with DCM-associated mutations including RBM20 (Wyles et al., 2016a), PLB (Karakikes et al., 2015), EMD (Shimojima et al., 2017), or CSRP3 (Li et al., 2019), showing marked calcium handling abnormalities that may recapitulate the early myocardial alterations occurring in human DCM hearts. However, the underlying molecular mechanisms of myocardial dysfunction and damage in the presence of DCM-related mutations in various genes can be completely different despite the common final phenotype.

**FIGURE 4:**
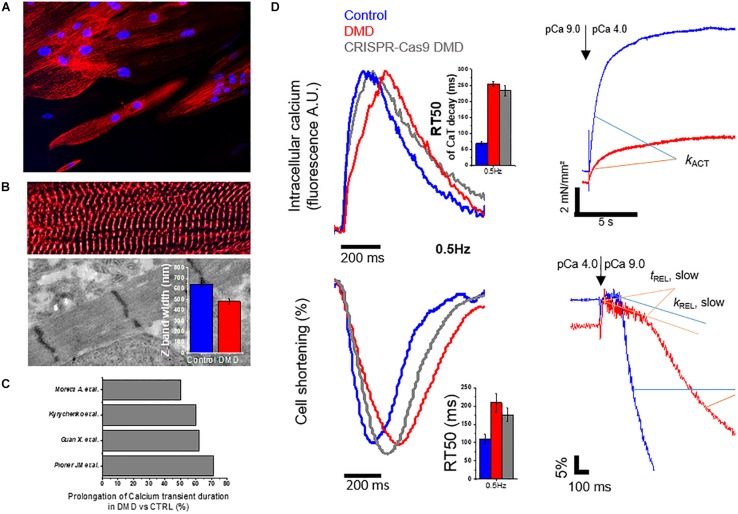
Morphology and function altered in DMD-hiPSC-CMs from patient confirmed in a CRISPR-Cas9 gene edited cell line. Original data are modified from Pioner et al. (2019b) with the correct permission from the owner (Pioner et al., 2019a). A DMD-hiPSC-cell line from a patient (with Δ Exon 50 in DMD gene) and a CRISPR-Cas9 gene edited cell line (c.263 ΔG) targeting Exon 1 in healthy control cell line (Control) were generated and the cardiomyocytes were matured onto nanotopographic cues for 3 months. **(A)** DMD-hiPSC-CMs displayed aspect ratio similar to adult cardiomyocytes and Z-bands were observed across the entire cell width (diameter), suggesting DMD-CMs experienced hypertrophy and a greater number of myofibrils in parallel. **(B)** Despite similar myofibril alignment to control-hiPSC-CMs, sarcomere diameters (estimated from the length of Z-bands by transmitted electron microscopy) were significantly smaller, suggesting a possible reduction in the parallel assembly of myofilaments within individual myofibrils. **(C)** Meta-analysis on Calcium transient duration. **(D)** Representative traces of calcium transients (CaT), cell shortening and myofibril mechanics of DMD-hiPSC-CMs compared to controls. Simultaneous recordings at single cell level revealed slower CaT decay (estimated from time to peak to 50% of CaT decay, RT50, ms), slower cell relaxation (RT50, ms) at 37°C and external pacing (0.5 Hz reported). Compared to other studies reporting similar analysis of calcium transients, slower CaT duration might be a peculiarity of DMD-hiPSC-CMs. Single myofibrils showed lower isometric-tension generating capacity and slower myofibril relaxation (slow *t*_*REL*_ and fast *k*_*REL*_). This study concluded that both calcium handling and myofibril abnormalities may contribute to prolong cell relaxation.

For DMD, two main hypotheses have been advanced: (1) membrane fragility or damage leading to membrane tear or rupture that facilitates abnormal calcium influx, as indirectly demonstrated by treating DMD cells with membrane sealants (i.e., Polaxamer 188) (Yasuda et al., 2005); (2) altered ion channel function and dysregulation of calcium homeostasis as a direct consequence of the altered dystrophin complex (Zhan et al., 2014). Guan and collaborators generated cardiomyocytes derived from the urine of a dystrophin-mutant DMD patient with a deletion of exon 50 (DMD-Δ exon 50), resulting in a dystrophin-deficient phenotype. DMD-Δexon50 displayed unique features such as increased membrane susceptibility to hypotonic stress, slower Ca^2+^ transients in the early phases after differentiation and increased probability of mitochondrial permeability pore opening (Guan et al., 2014). Similarly, DMD cell lines with deletion of exons 49–50 (DMD-Δ exons 49–50) overexpressed the immunoproteasome subunits, while their inhibition by ONX-0914 (proteasome inhibitor) reduced the sarcolemmal damage, as indicated by decreased release of cTnI and TNF-α after treatment (Farini et al., 2019). Lin and collaborators described hiPSC-CM cell lines from two patients carrying out-of-frame deletion in DMD exons 45–52 (DMD-Δ exons 45–52). They reported DMD-CMs with profound reduction of the L-type calcium current and increased cytosolic Ca^2+^ levels, and identified a downstream activation DIABLO-CASP3 that was related to mitochondrial apoptosis (Hinson et al., 2015). This mechanism is in keeping with the increased reactive oxygen species (ROS) levels found in DMD-CM models (Gartz et al., 2018; Jelinkova et al., 2019). The membrane sealant P188 (Polaxamer 188) was able to normalize the increased diastolic calcium levels and to revert the increase in the rate of apoptosis, supporting the hypothesis that membrane damage/fragility leads to intracellular calcium overload even in the early stages of myocardial differentiation (Lin et al., 2015). A unifying hypothesis may involve mechanosensitive channels, e.g., the transient receptor potential canonical channel 1 (TRPC1), which transduce membrane stretch into a cationic (Na^+^, K^+^, Ca^2+^, and Mg^2+^) flux across the sarcolemma, and may be gated by an excessive tension developed within the lipid bilayer, as previously reviewed (Maroto et al., 2005). In support of this idea, a recent test exposed DMD (DMD-Δ exons 43–45) and control hiPSC-CMs, plated on a fibronectin-coated silicon chamber, to a periodical mechanical stretching (120% elongation) at a frequency of 60 cycles per min and measured the response of intracellular calcium levels after 3 h (Tsurumi et al., 2019). A short-term mechanical stretch protocol was sufficient to increase diastolic calcium levels and calcium transient amplitude in DMD cells while it did not cause such effects in controls. However, these studies are limited by the small number of cell lines and by the use of immature cardiomyocytes in the early-phases of cardiac differentiation and maturation. We recently reported the analysis of calcium transients in hiPSC-CMs at day 100 of differentiation, comparing the DMD-Δ Exon 50 line to a healthy control and its CRISPR-Cas9 genome edited isogenic control (Exon 1 deletion in the DMD gene, DMD-c.263delG) (Pioner et al., 2019a; Figure 4). At this advanced stage of differentiation and maturation, hiPSC-CMs paced by field stimulation showed adaptation to frequency changes. DMD-Δ Exon 50 showed slower calcium transient rise and decay compared to controls, and these changes were comparable to those observed in our CRISPR-Cas9 generated cell line (DMD-c.263delG) expressing a shorter synthetic dystrophin. Slower duration of calcium transients is an alteration found in other DMD cell lines (Figure 4C). Both cell lines were associated with prolonged cardiomyocyte relaxation at low loading conditions (Figure 4D). This directly demonstrated that the lack of the full-length dystrophin is sufficient to perturb normal contractility of cardiomyocytes, with abnormal calcium homeostasis as a leading consequence. Moreover, another recent study generated a cell line from a male patient (DMD- c.5899C > T) and from a DMD female carrier (Δ Exons 8–12). Interestingly, both DMD cell lines showed prolonged action potential (AP) duration compared to control lines, and those measured in cells from the female carrier were even larger (APD90, 600 vs. 400 ms in the male patient). The prolongation of APs was attributed to an increase of L-type calcium (I_*Ca–L*_) peak current amplitude and CACNA1C expression (I_*Ca–L*_ main channel subunit), which were even more evident in the iPSC-CMs obtained from the female carrier. Regulation or expression of ion channels might be dependent on the specific protein defects rather than on the common downstream pathways related to cell degeneration toward a DCM phenotype. For instance, another DCM-hiPSC model was obtained from patients harboring a fukutin-related protein gene mutation (826C > A; Leu276Ile, transmembrane protein) associated to Limb-Girdle muscular dystrophy (LGMD) and was studied at comparable levels of maturation. In this case, the authors reported DCM-hiPSC-CMs with reduced AP amplitude and shorter AP duration compared to healthy controls, with reduced peak and late Na currents (I_*Na*_), as well as lower peak L-type calcium channel current; these changes were likely related to the reduced expression of their related genes, SCN5A and CACNA1C. In addition, the rapidly activating delayed rectifier potassium current (I_*Kr*_) was reduced, whereas the transient outward current (I_*to*_) and slowly activating delayed-rectifier potassium current (I_*Ks*_) were similar in DCM and control cardiomyocytes (El-Battrawy et al., 2018).

### Force and Contractile Properties of DMD-hiPSC Cardiomyocyte Models

The N-terminal domain of dystrophin has homology for cytoskeletal F-actin and interacts in the context of costamere domains that couple sarcolemma to sarcomeric Z-bands. As cardiac cells rapidly shorten during contraction, electrostatic interaction between the basic spectrin repeats and actin filaments serve to dampen elastic extension during stretch (Ervasti, 2007). In this context, a complex network involves multiple DCM-related proteins including the sarcomere proteins titin (TTN), β-myosin heavy chain (β-MHC), cTnT, cTnI, cTnC, tropomyosin cardiac troponin I (cTnI)–interacting kinase (TNNI3K) and other Z-band interacting proteins such as α-actinin, desmin, telethonin, cardiac ankyrin repeat protein, nebulette, α-B-crystallin and cipher. The Z-band complex seems to be involved in the mechanosensory mechanisms that convert mechanical stimuli into biochemical signals, and dystrophin has connections with several Z-band proteins. Consequently, DMD- with Δexon50 displayed less adaptiveness to variations of the topographical cues (cell and sarcomere alignment), which was related to reduced actin turnover, as studied by fluorescence recovery after photobleaching (FRAP). This behavior was reproduced in a DMD CRISPR-Cas9 gene-edited cell line (Macadangdang et al., 2015). By comparison, hiPSC-CMs with a mutation in the RBM20 gene exhibited heterogeneous sarcomeric organization patterns. RBM20-engineered heart tissues (EHTs) showed impaired force of contraction and increased passive tension that was associated to a lower degree of the switch from the N2BA to the N2B isoform of titin, as a consequence of the S635A RBM20 mutation impacting on RNA splicing and transcription (Streckfuss-Bomeke et al., 2017). In a different way, loss of dystrophin may impact both cytoskeletal F-actin and sarcomeric actin in cardiomyocytes.

Myofibrils are the most abundant organelles of adult cardiomyocytes, occupying approximately 50–60% of the cytoplasmic volume. However, myofibrils are less represented in the contractile machinery of hiPSC-CMs, in which the sarcomere bands and lines gradually form in the advanced stages of maturation (Gherghiceanu et al., 2011). In this context, we investigated whether loss of dystrophin may affect the contractile properties at single myofibril level by applying a cell skinning protocol followed by tissue homogenizationto obtain single myofibrils, which were studied using a custom-made force-recording apparatus with fast solution switching, as previously described (Pioner et al., 2016). We found reduced tension production upon Ca-mediated activation in DMD myofibrils, as compared to controls. However, the kinetics of force development (*k*_*ACT*_) and relaxation (slow *k*_*REL*_) after calcium removal were not different, suggesting no changes in the rate of crossbridge cycling during tension development and detachment. Also, we found a slower fast phase of relaxation (fast *k*_*REL*_), which is mainly regulated by inter-sarcomere dynamics (Pioner et al., 2019a; Figure 4D), paralleled by a slower contraction (shortening) of intact cells (Figure 4B). Altered myofibril contractile function could be related to developmental alterations, although the mechanism is not clear (Figure 4A). This should encourage future studies to better define the fundamental role of sarcomere protein composition for the definition of contractile properties. Another group investigated mutations in the dystrophin actin-binding domain (ABD1) causing truncated forms of dystrophin (DMD- Δ Exon 3–9, 6–9, or 7–11). Force measurements using multicellular preparations (engineered heart muscle, EHM) displayed lower force in DMD-mutant cell lines and the *de novo* insertion of the missing exon using CRISPR-Cas9 technology only partially restored the contractile performance. Among other DCM models, the hiPSC-CMs with absence/reduction of full-length dystrophin may contribute to evaluate the functional consequences of dystrophin loss and elucidate the role of a variety of proteins interacting around the N-terminal of full-length dystrophin, which are still elusive.

## Overcoming the Limitations of hiPSC-CMs: Patient-Specific Validation and High-Throughput Studies

To date, the possibility of using hiPSC-CMs to predict individual prognosis or response to therapies is limited by lack of direct validations, that is, patient-specific hiPSC-CMs have never been tested against actual adult cardiomyocytes from the same patient or from patients with a similar condition. This is due to the poor availability of human samples from patients with cardiomyopathies or other arrhythmogenic conditions, and the limited experience of many research centers in investigating fresh human myocardium and cardiomyocytes (Tardiff et al., 2015). We have recently developed a technique (Coppini et al., 2014) to isolate single ventricular cardiomyocytes and trabecule from the surgical samples of obstructive HCM patients. We were therefore able to study adult CMs in order to characterize the functional abnormalities of action potentials, ion currents, Ca^2+^ handling, myocardial contraction and relaxation, and test the effectiveness of different ion channel blockers such as ranolazine or disopyramide (Coppini et al., 2013; Coppini et al., 2018; Ferrantini et al., 2018). In Pioner et al. (2019b), we compared the physiological properties of iPSC-CMs from an healthy donor, differentiated using nanopatterned surfaces and long-term cultures, with those of adult cardiomyocytes from non-failing non-hypertrophic surgical patients (Pioner et al., 2019b). In terms of action potential shape and duration, calcium transient properties and response to β-adrenergic agonists, hiPSC-CMs at an advanced degree of maturation closely approximated the features of freshly isolated human adult cardiomyocytes (Pioner et al., 2019b). While obtaining fresh cardiac samples from DMD patients is hardly feasible, our technique can be developed to allow the isolation of cardiomyocytes from intramyocardial biopsies, to be used for functional studies in selected patients, allowing a direct comparison with individual hiPSC-CMs. Moreover, in order to improve hiPSC-CM maturation, single cardiomyocytes or 3D cultures (engineered heart tissues; Gupta et al., 2018) could be challenged during maturation using electrical stimulation at different pacing rates and periodic stretch-release protocols, simulating the contraction-relaxation cycle of the native heart (Sun and Nunes, 2017; Ronaldson-Bouchard et al., 2019). Such approach to cardiomyocyte differentiation might be easily achieved using smart contractile materials as culture substrates, such as liquid-crystal elastomer, which we recently developed and characterized (Martella et al., 2017; Ferrantini et al., 2019).

The possibility offered by hiPSC-CMs to identify patient- or mutation-specific mechanisms of disease and test novel therapies in a personalized manner is hampered by the fact that current approaches are extremely costly and time consuming. The development of rapid high-throughput assays to study the pathophysiological features of different hiPSC-CM lines and test novel therapeutic approaches is therefore warranted. We recently developed a system to simultaneously record action potentials and intracellular Ca^2+^ from multiple iPSC-CMs using a fast camera, paralleled by a streamlined data analysis system to rapidly extract the main functional biomarkers from each recording (Pioner et al., 2019b). In an attempt to further improve the throughput of cell-line analysis, a possible approach would be an all-optical semi-automatic platform combining the parallel detection of multiple functional properties with electrode-free optogenetic control of electrical function in the sample. This approach would be feasible using genetically encoded voltage indicators (GEVIs) or calcium indicators (GECIs) (Kaestner et al., 2014; Huebsch et al., 2015; Shaheen et al., 2018) and gene-encoded actuators (channelrhodopsin) (Klimas et al., 2016). Such comprehensive platform, when combined with a semi-automated experimental-control and analysis system (Klimas et al., 2016), will allow repeatable longitudinal prolonged evaluations of iPSC-CMs at different time points and facilitate the evaluation of their response to different treatments. In addition to these technologies, CRISPR-Cas9 gene repair was recently improved by the newly discovered CRISPR-Cpf1 nuclease (Zhang et al., 2017). CRISPR-Cpf1 is driven by a single RNA guide (gRNA) and prefers T-rich protospacer adjacent motif (PAM) sequences: this feature is important for the potential correction of other disease-related mutations, because not all mutation sites contain G-rich PAM sequences for SpCas9 or PAMs for other Cas9 orthologs. Importantly, Cpf1 is about 140 amminoacids smaller than the most widely used SpCas9, which can potentially enhance the packaging and delivery by adenovirus, adeno-associated (AAV) or other viral constructs. Finally, Cpf1 can be used to permanently correct *DMD* mutations, restoring dystrophin expression and efficiently preventing the progression of the disease, as reported in DMD patient-derived iPSCs and *mdx* mice. The combination of biophysical approaches with the breakthrough of increasingly precise genome editing techniques may pave the way to a truly personalized medicine for DMD patients.

## Translating Stem Cell Modeling From Bench to Bed Side

Four groups of molecules are currently in use in the clinical management of patients with DMD cardiomyopathy: renin-angiotensin-inhibiting agents, beta-adrenergic receptor blockers, mineralocorticoid receptor antagonists and corticosteroids. Although useful for symptom management, none of these molecules is capable of modifying the natural history of the disease. Currently, none of the stem cell-based studies have reported or suggested novel pharmacological approaches for dystrophin-associated cardiomyopathies. Few pilot tests on animal models were attempted, using a membrane sealant (Lin et al., 2015) or an immunoproteasome inhibitor (Farini et al., 2019), but these agents are not currently under development for use in patients. In other DCM models, a limited number of disease modifying therapeutic approaches were attempted, including the overexpression of Serca2a by viral-vector gene-therapy (Sun et al., 2012), and the modulation of SERCA/PLN through the mechanoreceptor protein integrin-linked kinase (ILK) (Traister et al., 2014). Indeed, most of the DCM hiPSC studies sought to identify novel mechanistic targets and were only labeled as a promising platform for a pharmacological therapy, but never used for an actual pharmacological validation. This is undoubtedly related to the immaturity of hiPSC-CM models and the fact that findings obtained using *in vitro* models cannot be so easily translated into trials *in vivo*.

In recent years, potentially curative approaches for DMD have begun to take shape. Unlike small molecule treatments, CRISPR-Cas9 or TALEN gene-targeted therapies are designed with the goal to induce expression of a functional gene product that will reestablish normal myocyte physiology. Since these therapies aim to repair the original defect in the cell by restoring functional dystrophin expression, their success is less dependent upon identifying the exact molecular consequences of the lack of dystrophin. As shown in Table 3, major efforts involved the use CRISPR-Cas9 in the design of mutation-specific therapies, which include exon skipping, reframe or deletion of portions in the DMD gene. Notably, successful restoration of contractile function was observed in a large group of hiPSC models carrying mutations in the ABD domain (mostly linked to BMD) or in the central domains (mostly associated to DMD) (Kyrychenko et al., 2017; Zhang et al., 2017; Long et al., 2018; Min et al., 2019). For this purpose, more effort should be moved toward the generation of BMD-patient cell lines (Gowran et al., 2018), as this patient group frequently diagnosed mid-life and is definitely an understudied group. In addition, BMD patients appear to be the most promising candidates for autologous cell transplantation as well as for *in vitro* studies aimed at identifying specific, novel targets that emerge in the presence of semi-functional truncated dystrophin proteins. Current strategies still lack the ability to obtain a lasting and efficient correction of the primary defect. In addition, DMD and BMD are associated with a variety of different mutations in the DMD gene. Therefore, a host of different therapeutic approaches are required for different patient subgroups, depending on the site and nature of their mutation. For instance, PTC124 (Ataluren) has been used in Europe to treat DMD patients carrying nonsense mutations (10% of total variants; Bladen et al., 2015). PTC124 renders ribosomes less sensitive to premature stop codons by promoting insertion of near-cognate tRNA at the site of nonsense codons with no apparent effects on downstream transcription, mRNA processing, or stability of the resulting protein, thereby allowing the production of functional dystrophin proteins. Ataluren works particularly well for the stop codon “UGA” (Welch et al., 2007). For this reason, the drug could be eligible for preclinical personalized tests on patient-specific stem cell-based models, which have been rarely attempted so far (Koopmann et al., 2012; Lee et al., 2017). Finally, innovative therapeutic approaches may consider repositioning drugs in use for other conditions or strategies acting on molecular factors that induce or downregulate common molecular pathways involved in genetic cardiomyopathies. Although such “universal” approaches will not restore dystrophin expression in the heart, they could still delay disease progression, be well tolerated, and be appropriate for a broader range of patients with DMD.

**TABLE 3:** Gene therapy strategies and application to hiPSC-CM models to restore dystrophin function.

Approach	Target mutation type	Dystrophin product	Strengths	Challenges	hiPSC-CM
Stop codon readthrough	Nonsense point mutations	Complete	• Well-tolerated (PTC124 or ataluren)	• *Low efficiency in the heart* • Low prevalence of amenable mutations • Frequent re-dosing	?
AON-mediated exon skipping	Frameshift mutations	Lacking existing deletion and additional exon(s)	• Well-tolerated • *Effective at cell level*	• Poor cardiac uptake of PMO • Frequent re-dosing • Low number of amenable mutations for each AON drug	• Efficacy • Reduced arrhythmias (Liang et al., 2019)
AAV micro-dystrophin	not interacting with endogenous gene)	Extensively truncated but functional	• *High efficacy in heart* • High efficacy in skeletal muscle • Lasting (multiple years)	• Potentially immunogenic • Potential for null effect with pre-existing immunity	?
CRISPR-Cas9	Frameshift, insertion, and nonsense mutations	Depends on editing strategy [ranging from complete to lacking deletion and additional exon(s)]	• *High efficacy in heart and skeletal muscle* • Versatile • Genomic correction is life-long (theoretically)	• Potentially immunogenic • *Risk of off-target editing* • Low number of amenable mutations for each CRISPR drug	• Efficacy • Restored contractile force of EHTs (Kyrychenko et al., 2017; Long et al., 2018; Min et al., 2019; Zhang et al., 2017)

## Conclusion

In this review, we analyzed the current insights of DMD-associated cardiomyopathy, from the clinical aspects to innovative disease modeling based on human iPSC derived cardiomyocytes, in the attempt to decipher specific mechanisms underlying DCM pathogenesis in DMD patients. Human models of iPSC-cardiomyocytes are a viable cell-based platform for investigating the human cardiac phenotype resulting from specific gene mutations. These models can recapitulate the facets of the physiology and pathophysiology of native cardiomyocytes, opening new opportunities for research. hiPSC-cardiomyocytes allow the study of unique individual patients without reliance on isogenic strains of animals and avoiding invasive biopsies of human cardiac tissue samples. Their progressive maturation of their cardiac phenotype, obtained with advanced differentiation techniques, is a powerful tool for investigating the early-stage consequences of dystrophin mutations that DMD, and their effects on cardiac development. Existing studies suggest that the pathogenic mechanisms appear early in the disease progression as a combination of the developmental consequences of the absence of full-length dystrophin (Dp427) in cardiomyocytes. Gene therapy with micro dystrophin, up-regulation of utrophin or exon skipping approaches are currently the most promising ways to rescue or at least mitigate the DMD phenotype. For this reason, despite the obvious limitations related mostly to their immature phenotype, the advent of hiPSC-cardiomyocytes presents a unique opportunity to improve our approach to the understanding of the pathophysiology of DMD cardiomyopathy.

## Author Contributions

JP, AF, and CF contributed to the design and wrote the manuscript in consultation with RC, LS, MD, SF, CP, and IO. JP and NC performed the meta-analysis and prepared figures and tables. All authors critically reviewed the manuscript and declared that the research was conducted in the absence of any commercial or financial relationships that could be construed as a potential conflict of interest.

## Conflict of Interest

The authors declare that the research was conducted in the absence of any commercial or financial relationships that could be construed as a potential conflict of interest.

## Abbreviations

AAV: Adeno-associated virus
*ABCC9*: Sulfonylurea receptor 2A, component of ATP-sensitive potassium channel
ABD1: actin-binding domain
*ACTC1*: α-cardiac actin
ACTN2: α-actinin 2
AF: atrial fibrillation
*ANKRD1*: Cardiac ankyrin repeat protein
*ARVC*: arrhythmogenic right ventricular cardiomyopathy
*BAG3*: BCL2-associated athanogene 3
BMD: Becker Muscular Dystrophy
CM: Cardiomyocytes
CMR: cardiac magnetic resonance
CRISPR-Cas9: Clustered Regularly Interspaced Short Palindromic Repeats
*CRYAB*: α-B-crystallin
*CSRP3*: Muscle LIM protein
DCM: Dilated cardiomyopathy
*DES*: Desmin
DGC: dystrophin glycoproteic complex
DMD: Duchenne Muscular Dystrophy
*DMD*: dystrophin gene
*DNAJC19*: HSP40 homolog, C19
Dp427: Full-length dystrophin isoform (427 kDa)
Dp71: Dystrophin isoform (71 kDa)
*DSP*: Desmoplakin
ECG: electrocardiogram
EHT: engineered heart tissue
*EMD*: Emerin
fast *k*_*REL*_: fast phase of myofibril relaxation
*FLNC*: Filamin C
HCM: hypertrophic cardiomyopathy
HF: Heart failure
hiPSC: Human induced pluripotent stem cell
HT: heart transplantation
ICD: implantable cardioverter defibrillators
*k*_*ACT*_: myofibril kinetic of force development
*KCNQ1*: Potassium channel
*LDB3*: Cipher/ZASP
*LMNA*: Lamin A/C
*LQT*: long QT syndrome
LV: left ventricle
LVEF: left ventricular ejection fraction
LVNC: left ventricular non-compaction
MF: myocardial fibrosis
*MYH7*: β-Myosin heavy chain
*NEBL*: Nebulette
nNOS: nitric oxide synthase
OMT: optimal medical therapy
PAM: protospacer adjacent motif
PLN: Phospholamban
PMO: phosphorodiamidate morpholino oligomer
*RBM20*: RNA-binding protein 20
RCM: restrictive cardiomyopathy
RCT: randomized control trial
SCD: sudden cardiac death
*SCN5A*: Type V voltage-gatedcardiac Na channel
SGCA, SGCB, SGCD: α-/β-/δ-Sarcoglycan
slow *k*_*REL*_: slow linear phase of myofibril relaxation
TALEN: transcription activator-like effector nucleases
*TAZ/G4.5*: Tafazzin
*TCAP*: Titin-cap/telethonin
*TNNC1*: Cardiac troponin C
*TNNI3*: Cardiac troponin I
*TNNI3K*: Troponin I–interactingkinase
*TNNT2*: Cardiac troponin T
*TPM1*: α-Tropomyosin
*TTN*: Titin
*VCL*: Metavinculin
VF: ventricular fibrillation
VT: ventricular tachycardia.
